# SP1-mediated up-regulation of lncRNA TUG1 underlines an oncogenic property in colorectal cancer

**DOI:** 10.1038/s41419-022-04805-w

**Published:** 2022-05-04

**Authors:** Wei Liu, Jin Meng, Rongjun Su, Changjun Shen, Shuai Zhang, Yantao Zhao, Wenqi Liu, Jiang Du, Shuai Zhu, Pan Li, Zhigang Wang, Xiaoxia Li

**Affiliations:** 1 Department of General Surgery, Yan’an People’s Hospital, Yan’an, 716000 P.R. China; 2 Department of Fifth Treatment Areas of Anorectal Disease, Shenyang Coloproctology Hospital, Shenyang, 110002 P.R. China

**Keywords:** Cancer, Diseases

## Abstract

The long non-coding RNA (lncRNA) taurine up-regulated gene 1 (TUG1) acts as tumor-promoting factor in colorectal cancer (CRC). We aimed to elucidate the mechanism by which the transcription factor specificity protein 1 (SP1) regulates TUG1 and microRNAs (miRs)/mRNAs in the context of CRC, which has not been fully studied before. Expression patterns of TUG1 and SP1 were determined in clinical CRC samples and cells, followed by identification of their interaction. Next, the functional significance of TUG1 in CRC was investigated. An in vivo CRC model was established to validate the effect of TUG1. The results demonstrated that TUG1 and SP1 were highly-expressed in CRC, wherein SP1 bound to the TUG1 promoter and consequently, positively regulated its expression. Silencing of TUG1 caused suppression of CRC cell growth and promotion of cell apoptosis. TUG1 could bind to miR-421 to increase KDM2A expression, a target gene of miR-421. TUG1 could activate the ERK pathway by impairing miR-421-targeted inhibition of KDM2A. Additionally, SP1 could facilitate the tumorigenesis of CRC cells in vivo by regulating the TUG1/miR-421/KDM2A/ERK axis. Altogether, the current study emphasizes the oncogenic role of TUG1 in CRC, and illustrates its interactions with the upstream transcription factor SP1 and the downstream modulatory axis miR-421/KDM2A/ERK, thus offering novel insights into the cancerogenic mechanism in CRC.

## Introduction

Colorectal cancer (CRC) represents a significant proportion of the total disease burden attributed to malignancies all over the world [1]. Current treatment approaches for patients with primary and metastatic CRC include the use of laparoscopic surgery for primary cases, more-aggressive resection for metastatic tumors, and radiotherapy for rectal neoplasm along with neoadjuvant and palliative chemotherapies [2]. Despite tremendous improvement in antineoplastic therapies, the five-year survival rates for CRC remain dismal, whereas high drug prices impose a great burden on the total economic cost of CRC [3]. Increasing numbers of investigations are addressing the identification of biomolecules implicated with the pathophysiological events of CRC, in hopes of developing new therapeutic targets for better treatment [4, 5].

Interestingly, a set of non-coding RNAs (ncRNAs) comprising of microRNAs (miRNAs) and long non-coding RNAs (lncRNAs) are emerging as novel biomarkers for the detection and therapeutics of CRC [6]. More specifically, a number of studies have documented a tumor-promotive role of TUG1 in several malignancies such as cervical cancer [7], prostate cancer [7], and especially CRC [8]. TUG1 exerts its tumor-promotive function *via* diverse mechanisms including RNA-RNA interactions [9] and the Wnt/β-catenin pathway [10]. However, the significance of TUG1 in CRC remains largely undefined.

Meanwhile, transcription factors, known to mediate chromatin alteration and gene transcription by recognizing specific DNA sequences, have also been implicated in various aspects of human physiological processes and diseases [11]. Moreover, the deregulation of transcription factors is induced in various malignancies directly *via* translocation of chromosomes, amplification or deletion of genes, and site-specific mutagenesis, or indirectly by means of mutations in non-coding DNAs [12]. One such member of the nuclear transcription factor family, SP1 was recently identified to cause an elevation in TUG1 in hepatocellular carcinoma [13]. Additional investigations on TUG1 have also highlighted its ability to mediate the apoptosis of lens epithelial cells by negatively-regulating miR-421 [14], a tumor-suppressive miR in CRC [15]. Inherently, miRNAs are small endogenous ncRNAs that modulate gene expressions at a post-transcriptional level *via* binding to the 3’UTR of specific target mRNAs [16]. Furthermore, online prediction databases indicate KDM2A, an oncogenic gene in CRC [17], to be a target of miR-421. Consequently, we tried to investigate whether SP1, miR-421, and KDM2A interacted with TUG1 to promote cancerogenesis in CRC, hoping to uncover novel therapeutic targets against CRC.

## Material and methods

### Ethics statement

All experimentation protocols were approved by the Ethics Committee of Yan’an People’s Hospital and performed in strict accordance with the Declaration of Helsinki. All participants signed informed consent documentation before sample collection. Animal experiments were implemented in the light of the International Guidelines of Animal Care and Use issued by the USA National Institutes of Health.

### Clinical tissue samples

A total of 20 patients, who were histologically confirmed with CRC at the Yan’an People’s Hospital from January 2012 to December 2015, were chose for our study. Patients receiving radiotherapy or chemotherapy prior to surgery were excluded. During surgery, CRC tissues and adjacent normal mucosal tissues (in the visible normal intestinal region, 10 cm away from cancer tissues) were obtained from all patients, and stored at −80 °C for subsequent analysis [18]. All the collected tissues were pathologically diagnosed by two independent pathologists.

### Cell culture and plasmid transfection

Human normal colonic epithelial cell line FHC (ATCC® CRL-1831), as well as human CRC cell lines HCT116 (ATCC® CCL-247™, Rockville, MD) and SW480 (ATCC® CCL-228™), was procured from ATCC (Manassas, VA). HCT116 cells were cultured in McCoy’s 5a medium containing 10% FBS (ATCC, No. 30-2007), while SW480 cells were maintained in Leibovitz’s L-15 medium (ATCC, No. 30-2008) containing 10% FBS. Meanwhile, FHC cells were cultured in DMEM/F12 medium. All the aforementioned cells were cultured in a humid incubator at 37 °C with 5% CO_2_ in air.

Subsequently, the cells were seeded into a six-well plate (3 × 10^5^ cells/well). Upon reaching 50% cell confluence, transfection was carried out using Lipofectamine 2000 reagent (11668-019, Invitrogen, Carlsbad, CA). siRNAs targeting SP1 (si-SP1-1, si-SP1-2), siRNAs targeting TUG1 (si-TUG1-1, si-TUG1-2), siRNA targeting KDM2A (si-KDM2A) and its negative control (si-NC), miR-421 mimic, mimic NC, TUG1 over-expression plasmid (oe-TUG1) and oe-NC were all synthesized and provided by GenePharma (Shanghai, China). The siRNA sequences are shown in Supplementary Table 1.

### Reverse transcription quantitative polymerase chain reaction

TRIzol reagent (10296028, Invitrogen) was utilized for total RNA extraction. A total of 5 µg of reverse transcriptase enzyme was utilized for synthesizing cDNA from the obtained RNA using SuperScript III RT kits (11752050; ABI-Invitrogen). For miRNA detection, with cDNA serving as a template, Reverse transcription quantitative polymerase chain reaction (RT-qPCR) was performed using TaqMan MicroRNA Assay. U6 was employed as the internal control for miRNA. For mRNA detection, RT-qPCR was performed following TaqMan Gene Expression Assays protocol (Applied Biosystems, Foster City, CA) with GAPDH serving as the internal reference. The relative quantification method (2^–△△CT^ method) was utilized to calculate the relative expression of the gene of interest. The primers are listed in Supplementary Table 2.

### Western blot assay

Total protein was extracted, electrophoresed and then electroblotted to polyvinylidene fluoride membranes (Amersham, Piscataway, NJ), which were blocked with 5% skim milk powder for 1 h. After that, the membrane was incubated overnight at 4°C with the following primary antibodies to SP1 (ab227383, 1: 5000), cleaved PARP (ab32064, 1: 5000), cleaved Caspase-3 (ab49822, 1: 500), KDM2A (ab191387, 1: 1000), p-ERK (ab223500, 1: 400), ERK (ab17942, 1: 1000), and β-actin (ab179467, 1: 5000) as well as with horseradish peroxidase (HRP)-labeled secondary antibody goat anti-rabbit IgG (H&L, ab6721, 1: 3000) for 1 h at room temperature. After scanning and development with an optical luminometer (General Electric, Waukesha, WI), the gray value of the target band was quantified using the Image Pro Plus 6.0 software (Media Cybernetics). All the above-mentioned antibodies were purchased from Abcam (Cambridge, UK).

### Luciferase assay

According to the analysis results from UCSC (http://genome.ucsc.edu/) and JASPAR (http://jaspar.genereg.net/), binding sites between SP1 protein and TUG1 were gained. Recombinant luciferase reporter gene vector was co-transfected with oe-SP1 into Hs 675.T, DiFi cells. Renilla luciferase expression vector pRL-TK (TaKaRa, Dalian, China) was utilized as an internal control. Subsequently, the luciferase activity was detected using a dual-luciferase detection kit (K801-200, Biovision) in a dual luciferase reporter gene analysis system (Promega, Madison, WI, USA).

TUG1 3’UTR or KDM2A wild-type (WT) or mutant (MUT) sequences were synthesized and cloned into the pMIR-reporter in the light of the luciferase detection kit (Promega). Using Lipofectamine 2000 reagent, Hs 675.T cells were co-transfected with WT or MUT plasmids and miR-421 mimic or mimic NC. After 48-h of transfection, the cells were harvested for luciferase activity detection by means of a dual luciferase assay (Promega).

### Chromatin immunoprecipitation

ChIP was implemented based on the previous study [19] with the rabbit IgG antibody (ab109489, 1: 300, Abcam) and the SP1-specific antibody (ab227383, 1: 200, Abcam). The enrichment of SP1 in the TUG1 promoter was detected using qPCR.

### CCK-8 assay

The cells were plated in 96-well plates (1 × 10^4^ cells per well). Cell transfection was performed the following day, and the cells were cultured for 0, 1, 2, and 3 d. The cells were then cultured with 10 μL of CCK-8 for another 2 h. Measurement of the absorbance 450 nm was implemented to determine cell viability.

### Transwell assay

The extracellular matrix (ECM) gel was pre-cooled at 4 °C overnight and diluted with serum-free medium (1:9). Next, 40 μL of ECM gel was added to the apical chamber of a Transwell chamber, and incubated at 37 °C with 5% CO_2_ in air for 5 h. After another round of incubation with DMEM (70 μL/chamber) for 0.5 h, cells starved for 24 h were resuspended in FBS-free DMEM to a final concentration of 2.5 × 10^5^/mL, whereupon 0.2 mL cell suspension was added to the apical chamber. Meanwhile, 700 μL of pre-chilled DMEM containing 10% FBS was added to the basolateral chamber. After 24 h of incubation at 37°C with 5% CO_2_ in air, the non-invading cells were wiped off with cotton balls, while the invaded cells were fixed with methanol for 30 min and stained with 0.1% crystal violet for 20 min. Finally, the invaded cells in randomly selected five visual fields were photographed and counted under an inverted microscope.

### Flow cytometric analysis

Cells at the logarithmic phase of growth were seeded in a six-well plate (2 × 10^5^/well), and cultured for 48 h. The cells were rinsed twice with pre-chilled PBS, and centrifuged at 4000 × *g* for 3 min. Next, the precipitate was stained with 400 μL of Annexin V-FITC on ice for 15 min and with 10 μL of PI for 5 min under dark conditions. Cell apoptosis was detected using a flow cytometer at 488 nm.

### RNA pull-down assay

Biotin-labeled RNA was transcribed with Biotin RNA Labeling Mix (Roche) and T7 RNA polymerase (Promega), treated with RNase-free DNase I (Promega), and then purified with RNeasy Mini Kit (QIAGEN). Afterwards, 5 g biotinylated RNA was heated at 95 °C for 5 min and allowed to stand on ice for 5 min and then at room temperature for 20 min. Next, the folded RNA was mixed with the cell lysate for 2 h, which was then incubated with 50 µL streptavidin agarose Beads (Invitrogen) for 1.5 h. Later, the beads were eluted and treated with ribonuclease, followed by dissolution in SDS buffer. The extracted proteins were measured using western blot assay.

### RNA immunoprecipitation

The binding of TUG1 to miR-421 was detected with the help of RIP kits (Merck Millipore, Billerica, MA, USA) [20, 21] with anti-Ago2 (ab32381, Abcam) and IgG antibody (ab172730, 1:1000, Abcam) as a NC.

### In vivo tumor formation model

A total of 48 female BALB/c nude mice (aged 3–4 weeks and weighing [16 ± 2 g]) were housed in a sterile environment at constant temperature (25–27 °C) and humidity (45–50%). The Hs 675.T cells stably transfected with sh-NC, sh-SP1, sh-SP1 + oe-NC, and sh-SP1 + oe-KDM2A were dispersed into a cell suspension (1 × 10^7^ cells/mL). Subsequently, 200 μL of cell suspension was subcutaneously injected into nude mice (*n* = 12 for mice following each treatment). The tumor volume was monitored and recorded every week. After 35 days, the nude mice were euthanized and the tumors were extracted for subsequent RT-qPCR and immunohistochemical analysis. The weight of the xenograft tumors was also weighed and recorded.

### Immunohistochemical analysis

The extracted mouse tumor tissues were fixed with 4% paraformaldehyde, embedded in paraffin and sliced into sections. Next, the sections were heated at 50 °C for 2 h, dewaxed with xylene, rehydrated with gradient ethanol, heated in sodium citrate buffer (10 mM sodium citrate, 0.05% Tween-20, pH 6.0) for 15–20 min, blocked with goat serum for 20 min, and probed with primary antibody to KDM2A (ab191387, 1: 250) at room temperature for 1 h, followed by incubation with 50 μL secondary antibody of goat anti-rabbit IgG (ab6721, 1: 1000) for 1 h. After that, the sections were reacted with streptavidin-peroxidase at 37 °C for 30 min, followed by development with DAB for 5–10 min. The sections were then counter-stained with hematoxylin for 2 min, and differentiated in hydrochloric acid (0.1 M). After rinsing for 10 min, the sections were dehydrated, cleared and sealed for microscopic examination.

### Statistical analysis

Statistical analyses were implemented using SPSS 21.0 statistical software (IBM Corp., Armonk, NY, USA). Measurement data were expressed as mean ± standard deviation. Data comparison in tissues was implemented by means of paired *t*-test, while that between the other two groups was conducted by means of unpaired *t*-test. Data among multiple groups were tested by one-way analysis of variance (ANOVA) with Tukey’s post-hoc test, and those at different time points were compared by two-way ANOVA with Bonferroni *post-hoc* test. Enumeration data were expressed by ratio or percentage and analyzed utilizing *χ*^2^ test. A value of *p* < 0.05 was indicative of statistical significance.

## Results

### SP1 and TUG1 are elevated in CRC and SP1 increases the expression of TUG1 by binding to the promoter region of TUG1

**Fig. 1:**
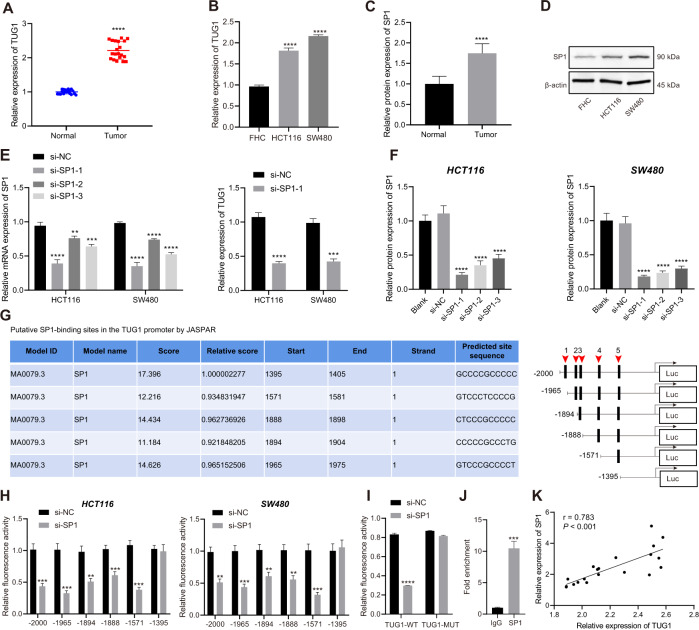
SP1 and TUG1 are abundant in CRC and SP1 up-regulates the expression of TUG1 by binding to the promoter region of TUG1. **A** TUG1 expression in the CRC and adjacent normal tissues (*n* = 20) determined by RT-qPCR. **B** TUG1 expression in normal human colonic epithelial cell line FHC and CRC cell lines HCT116 and SW480 determined by RT-qPCR. **C** SP1 protein level in the CRC and adjacent normal tissues (*n* = 20) measured by Western blot assay. **D** SP1 protein level in the human normal colonic epithelial cell line FHC and CRC cell lines HCT116 and SW480 measured by Western blot assay. **E** SP1 mRNA expression in the HCT116 and SW480 cells transfected with si-SP1-1, si-SP1-2 and si-SP1-3 determined by RT-qPCR. **F** SP1 protein level in the HCT116 and SW480 cells transfected with si-SP1-1, si-SP1-2, and si-SP1-3 measured by western blot assay. **G** The binding sites of SP1 in the TUG1 promoter region predicted by the JASPAR website. **H** After truncation of binding sites, the specific binding site 5 of SP1 in the TUG1 promoter region was determined by dual-luciferase reporter assay. **I** The binding of SP1 to the TUG1 promoter region with site 5 mutated analyzed by dual-luciferase reporter assay. **J** Binding of SP1 to the TUG1 promoter region at site 5 confirmed by ChIP assay. **K** Correlation between SP1 expression and TUG1 expression in clinical CRC tissue samples (*p* = 0.016). ^*^p < 0.05, ^**^p < 0.01, ^***^p < 0.001 and ^****^p < 0.0001, compared with adjacent normal tissues, FHC cell line, or HCT116 and SW480 cells transfected with si-NC or incubated with anti-IgG antibody. The measurement data are expressed as mean ± standard deviation. The experiments were repeated three times independently. Comparison between CRC tissues and adjacent normal tissues was conducted using paired *t*-test while between the other two groups by unpaired *t*-test. Multi-group comparison was conducted by one-way ANOVA with Tukey’s post hoc test.

First, we determined the expression pattern of TUG1 in 20 CRC tissues and normal tissues using RT-qPCR, which revealed an up-regulation of TUG1 in CRC tissues (Fig. 1A). Subsequently, with median TUG1 expression as the cut-off value, the patients with CRC were grouped into a high TUG1 expression group and a low TUG1 expression group, so as to analyze the correlation between TUG1 expression and the pathological characteristics of CRC patients. The results showed that TUG1 expression was correlated with tumor size, tumor node metastasis (TNM) stage, and lymph node metastasis (LNM), not with the age and gender (Supplementary Table 3). In addition, RT-qPCR results suggested higher TUG1 expression in human CRC cell lines HCT116 and SW480 compared to the human normal colonic epithelial cell line FHC (Fig. 1B). A previous report has indicated SP1 as an upstream mediator of TUG1 expression in hepatocellular carcinoma [13], but the significance of this interaction in regard to CRC is unknown. Here, the results of western blot assay demonstrated that SP1 protein levels were notably higher in CRC tissues and CRC cell lines HCT116 and SW480 relative to adjacent normal tissues and FHC cell line, respectively (Fig. 1C, D and Supplementary Fig. 1A).

To further identify whether SP1 regulates TUG1 expression, we silenced SP1 in HCT116 and SW480 cells using siRNAs, and confirmed the silencing efficiency by conducting RT-qPCR and western blot analysis. The results showed that si-SP1-1 exhibited the superior silencing efficiency, and was thus selected for subsequent experiments (Fig. 1E, F and Supplementary Fig. 1B). Additionally, RT-qPCR results presented a reduction in the TUG1 expression in HCT116 and SW480 cells following SP1 silencing (Fig. 1E).

The possible binding sites of SP1 in the TUG1 promoter region were predicted by the JASPAR website (Fig. 1G), and truncated one by one, whereupon a luciferase assay was conducted to confirm their binding relationship. After truncating the site 5, it was found that SP1 could not affect the luciferase activity of the TUG1 promoter, while truncating other binding sites did not alter the binding of SP1 to TUG1 promoter (Fig. 1H). On the other hand, SP1 silencing was observed to reduce the luciferase activity of TUG1-WT promoter sequence at site 5, but luciferase activity of MUT sequence with mutated site 5 remained unaffected (Fig. 1I). These findings suggested that SP1 could bind to the TUG1 promoter at site 5, and the results of subsequent Chromatin immunoprecipitation (ChIP) assay also validated this phenomenon (Fig. 1J). Finally, RT-PCR revealed a positive correlation between SP1 and TUG1 expression in 20 CRC tissues (Fig. 1K). The aforementioned data indicated that SP1 and TUG1 were up-regulated in CRC and that SP1 bound to the TUG1 promoter region to promote TUG1 expression.

### Loss of TUG1 inhibits the viability and invasion of CRC cells while promoting their apoptosis

**Fig. 2:**
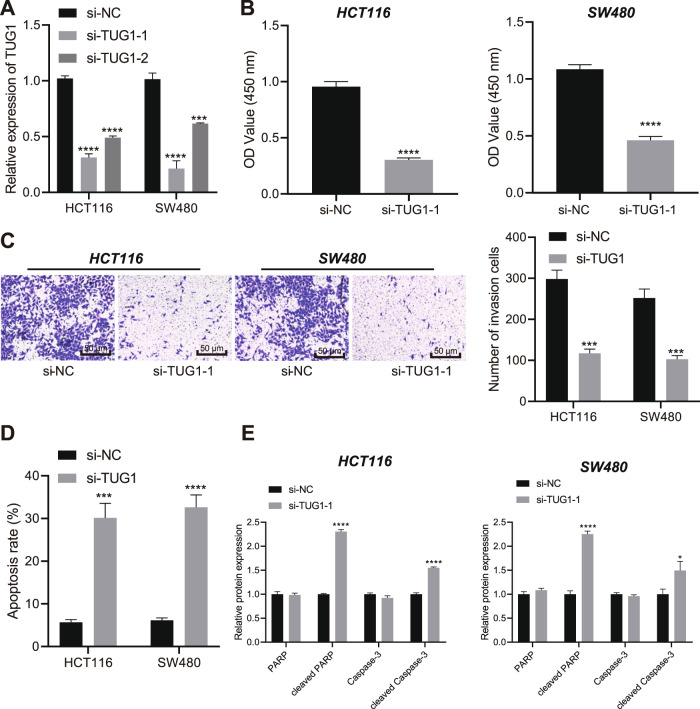
TUG1 loss-of-function leads to suppression of CRC cell viability and invasion and promotion of cell apoptosis. **A** Determination of TUG1 expression in HCT116 and SW480 cells transfected with si-TUG1-1 and si-TUG1-2 by RT-qPCR. **B**–**D** Assessment of HCT116 and SW480 cell viability (**B**), invasion (**C**), and apoptosis (**D**) by CCK-8 assay, Transwell assay (200×) and flow cytometry, respectively following transfection with si-TUG1-1. **E** Western blot assay of PARP, cleaved PARP, Caspase-3, and cleaved Caspase-3 proteins in HCT116 and SW480 cells following transfection with si-TUG1-1. ^*^p < 0.05, ^**^p < 0.01, ^***^p < 0.001 and ^****^p < 0.0001, compared with HCT116 and SW480 cells transfected with si-NC. The measurement data are expressed as mean ± standard deviation. The experiments were repeated three times independently. Comparison between two groups was conducted by unpaired *t*-test. Multi-group comparison was conducted by one-way ANOVA with Tukey’s post hoc test.

After uncovering the aberrant up-regulation of TUG1 in CRC cells, we knocked-down TUG1 in HCT116 and SW480 cells using siRNAs in order to probe into its effect on CRC cell behaviors. The transfection efficiency was determined by RT-qPCR, and si-TUG1-1 with the better efficiency was therefore used for the subsequent experiments (Fig. 2A). Additionally, TUG1 silencing resulted in suppressed cell viability (Fig. 2B) and invasion (Fig. 2C), yet increased apoptosis rate (Fig. 2D). Moreover, western blot assay demonstrated elevation in the protein levels of cleaved PARP and cleaved Caspase-3, while the total protein levels of PARP and Caspase-3 were unchanged in the HCT116 and SW480 cells transfected with si-TUG1-1 (Fig. 2E and Supplementary Fig. 2).

### TUG1 inhibits the expression of miR-421 to elevate the expression of KDM2A

**Fig. 3:**
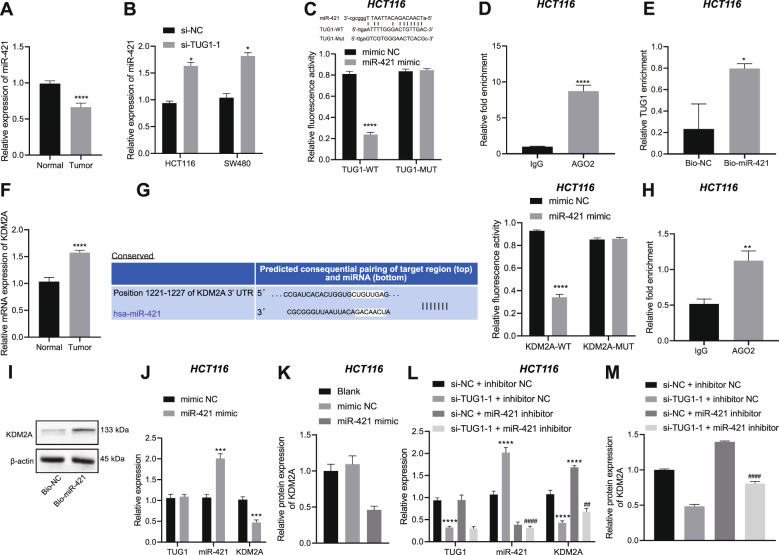
TUG1 positively regulates KDM2A by binding to miR-421 in HCT116 cells. **A** Determination of miR-421 expression in the CRC and adjacent normal tissues (*n* = 20) by RT-qPCR. **B** Determination of miR-421 expression in HCT116 and SW480 cells transfected with si-TUG1-1 by RT-qPCR. **C** The binding of TUG1 to miR-421 in HCT116 cells verified by dual-luciferase reporter assay. **D** The binding of TUG1 to miR-421 in HCT116 cells tested by RIP assay in combination with qPCR. **E** The binding of TUG1 to miR-421 in HCT116 cells tested using RNA pull-down. **F** Determination of KDM2A expression in the CRC and adjacent normal tissues (*n* = 20) by RT-qPCR. **G** The binding between miR-421 and KDM2A in HCT116 cells identified using dual-luciferase reporter assay. **H** The binding of miR-421 to KDM2A in HCT116 cells tested by RIP assay in combination with qPCR. **I** The binding of miR-421 to KDM2A in HCT116 cells tested using RNA pull-down. **J** Determination of TUG1, miR-421 and KDM2A expression in HCT116 cells transfected with miR-421 mimic by RT-qPCR. **K** Measurement of KDM2A protein level in HCT116 cells transfected with miR-421 mimic by western blot assay. **L** Determination of TUG1, miR-421 and KDM2A expression in HCT116 cells transfected with si-TUG1-1 or combined with miR-421 inhibitor by RT-qPCR. **M** Measurement of KDM2A protein level in HCT116 cells transfected with si-TUG1-1 or combined with miR-421 inhibitor by western blot assay. ^*^p < 0.05, ^**^p < 0.01, ^***^p < 0.001 and ^****^p < 0.0001, compared with adjacent normal tissues, HCT116 cells transfected with si-NC, mimic NC or si-NC + inhibitor NC, or HCT116 cells incubated with IgG or Bio-NC. ^#^*p* < 0.05, ^##^*p* < 0.01, and ^####^*p* < 0.0001, compared with HCT116 cells transfected with si-TUG1-1 + inhibitor NC. The measurement data are expressed as mean ± standard deviation. The experiments were repeated three times independently. Comparison between two groups was conducted by unpaired *t*-test. Multi-group comparison was conducted by one-way ANOVA with Tukey’s post hoc test.

We then proceeded to examine the mechanism of TUG1 in CRC. We discovered reduced miR-421 expression in CRC tissues (Fig. 3A). In addition, miR-421 expression was found to be up-regulated in HCT116 and SW480 cells transfected with si-TUG1-1 (Fig. 3B). Luciferase assay was then conducted to identify the interaction between those two RNAs using the TUG1-WT sequence containing the putative miR-421 binding site. As expected, it was found that transfection with miR-421 mimic in HCT116 and SW480 cells reduced the luciferase activity of TUG1-WT, without altering that of TUG1-MUT (Fig. 3C and Supplementary Fig. 3A). Meanwhile, findings from both RNA immunoprecipitation (RIP) (Fig. 3D and Supplementary Fig. 3B) and RNA pull-down (Fig. 3E and Supplementary Fig. 3C) assays also validated the binding of TUG1 to miR-421.

Online bioinformatics website predicted that miR-421 may bind to the histone demethylase KDM2A (Fig. 3G). We observed higher expression of KDM2A in CRC tissues than adjacent normal tissues (Fig. 3F). Moreover, the results of dual-luciferase reporter assay (Fig. 3G and Supplementary Fig. 3D), RIP assay (Fig. 3H and Supplementary Fig. 3E) and RNA pull-down assay (Fig. 3I and Supplementary Fig. 3F) verified the binding of miR-421 to KDM2A mRNA. Furthermore, transfection with miR-421 mimic in HCT116 and SW480 cells led to increased miR-421 expression yet decreased KDM2A expression (Fig. 3G, H, J, K and Supplementary Figs. 3G, H and 4A).

In addition, HCT116 and SW480 cells were then transfected with si-TUG1-1 or combined with miR-421 inhibitor. miR-421 expression was elevated and KDM2A expression was reduced in response to TUG1 knockdown, the effect of which was reversed by concomitant knockdown of TUG1 and miR-421 (Fig. 3L, M and Supplementary Figs. 3I, J and 4B). The above-mentioned results suggested that TUG1 down-regulated miR-421 expression by binding to miR-421, thereby increasing the expression of KDM2A.

### Knockdown of miR-421 elevates the expression of KDM2A, thus inducing the growth and invasion of CRC cells while arresting their apoptosis

**Fig. 4:**
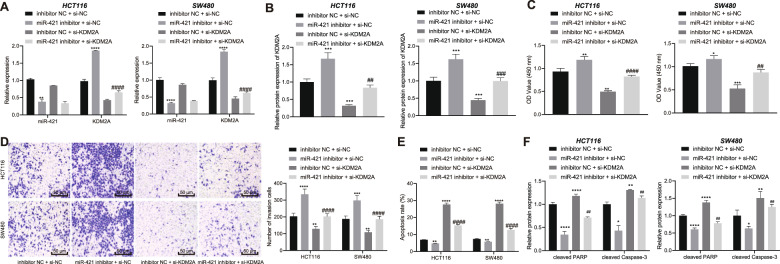
Knockdown of miR-421 enhances the expression of KDM2A to facilitate the growth and invasion of CRC cells and inhibit their apoptosis. HCT116 and SW480 cells were transfected with miR-421 inhibitor, si-KDM2A or both. **A** Determination of miR-421 and KDM2A expression in HCT116 and SW480 cells by RT-qPCR. **B** Western blot assay for KDM2A protein level in HCT116 and SW480 cells. **C**–**E** CCK-8 assay, Transwell assay (200×) and flow cytometry for HCT116 and SW480 cell viability (**C**) invasion (**D**), and apoptosis (**E**). **F** Western blot assay of cleaved PARP and cleaved Caspase-3 proteins in HCT116 and SW480 cells. ^*^p < 0.05, ^**^p < 0.01, ^***^p < 0.001, and ^****^p < 0.0001 compared with HCT116 cells transfected with inhibitor NC + si-NC. ^##^*p* < 0.01, and ^####^*p* < 0.0001, compared with HCT116 cells transfected with miR-421 inhibitor + si-NC. The measurement data are expressed as mean ± standard deviation. The experiments were repeated three times independently. Multi-group comparison was conducted by one-way ANOVA with Tukey’s post hoc test.

Since our initial findings suggested a regulatory axis consisting of TUG1/miR-421/KDM2A in CRC cells, we therefore speculated that this axis might exert a regulatory role in CRC cellular functions. HCT116 and SW480 cells were transfected with miR-421 inhibitor, si-KDM2A or both. We discovered that miR-421 expression was appreciably decreased and KDM2A expression was increased following treatment with miR-421 inhibitor. However, in the absence of KDM2A, KDM2A expression was diminished. In addition, concomitant knockdown of miR-421 and KDM2A reversed the promoting effect of miR-421 inhibitor on the KDM2A expression (Fig. 4A, B and Supplementary Fig. 5A).

Cell function assessment using CCK-8 (Fig. 4C), Transwell (Fig. 4D), and flow cytometry (Fig. 4E) provided evidence that miR-421 inhibition contributed to an enhancement in the viability and invasive capabilities of HCT116 and SW480 cells in addition to suppression of cell apoptosis, while these changes were all counteracted by transfection with si-KDM2A or miR-421 inhibitor + si-KDM2A. Simultaneously, cleaved PARP and cleaved Caspase-3 protein levels were quantified with western blot assay, which revealed that cleaved PARP and cleaved Caspase-3 protein levels were reduced by miR-421 inhibition, whereas these reductions were neutralized by si-KDM2A or miR-421 inhibitor + si-KDM2A (Fig. 4F and Supplementary Fig. 5B).

### Exacerbated in vitro progression of CRC through TUG1-induced ERK pathway activation

**Fig. 5:**
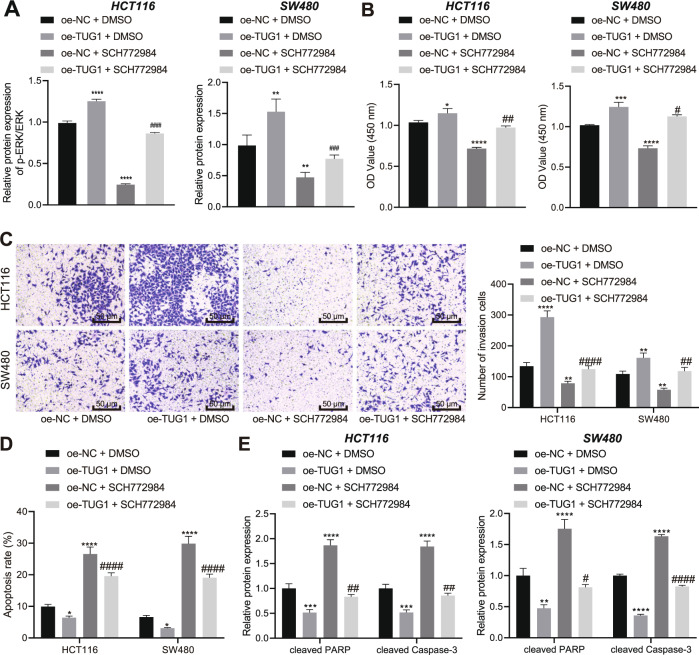
TUG1 contributes to ERK pathway activation to potentiate the malignant phenotypes of CRC cells. HCT116 and SW480 cells were transfected with oe-TUG1 and treated with ERK1/2 inhibitor SCH772984 or in combination. **A** Western blot assay of p-ERK and ERK protein levels in HCT116 and SW480 cells. **B**–**D** CCK-8, Transwell (200×) and Flow cytometric analyses for the viability (**B**) invasion (**C**) and apoptosis of HCT116 and SW480 cells (**D**). **E** Western blot assay for cleaved PARP and cleaved Caspase-3 proteins in HCT116 and SW480 cells. ^*^p < 0.05, ^**^p < 0.01, ^***^p < 0.001, and ^****^p < 0.0001, compared with HCT116 and SW480 cells treated with oe-NC + DMSO. ^#^*p* < 0.05, ^##^*p* < 0.01, ^###^*p* < 0.001, and ^####^*p* < 0.0001, compared with HCT116 and SW480 cells treated with oe-TUG1 + DMSO. The measurement data are expressed as mean ± standard deviation. The experiments were repeated three times independently. Multi-group comparison was conducted by one-way ANOVA with Tukey’s post hoc test.

ERK activation is regarded as a critical process for oxidative stress-stimulated invasiveness of CRC cells [21]. During further experimentation, we employed the ERK1/2 inhibitor SCH772984 (HY-50846, 10 μM; MedChemExpress) to block ERK pathway activation in HCT116 and SW480 cells over-expressing TUG1, so as to investigate whether TUG1 could regulate the ERK pathway. We found that TUG1 over-expression enhanced the ratio of pERK/ERK while SCH772984 treatment or combined with TUG1 over-expression decreased the ratio of pERK/ERK (Fig. 5A).

Furthermore, the results of CCK-8 (Fig. 5B) and Transwell assays (Fig. 5C) showed that the viability and invasion of HCT116 and SW480 cells were increased after over-expression of TUG1, whereas these effects were reversed by SCH772984 treatment or combined with TUG1 over-expression. In addition, TUG1 over-expression attenuated cell apoptosis while an opposite result was noted following SCH772984 treatment or combined with TUG1 over-expression (Fig. 5D). Meanwhile, the levels of the pro-apoptotic proteins cleaved PARP and cleaved Caspase-3 were reduced in HCT116 and SW480 cells over-expressing TUG1 while they were restored upon SCH772984 treatment or combined with TUG1 over-expression (Fig. 5E and Supplementary Fig. 6). Together, these results revealed that TUG1 functioned as an ERK pathway activator to facilitate the CRC progression in vitro.

### SP1 inhibits miR-421 and increases KDM2A by up-regulating TUG1, thereby activating the ERK pathway and promoting the tumorigenesis of CRC cells in vivo

**Fig. 6:**
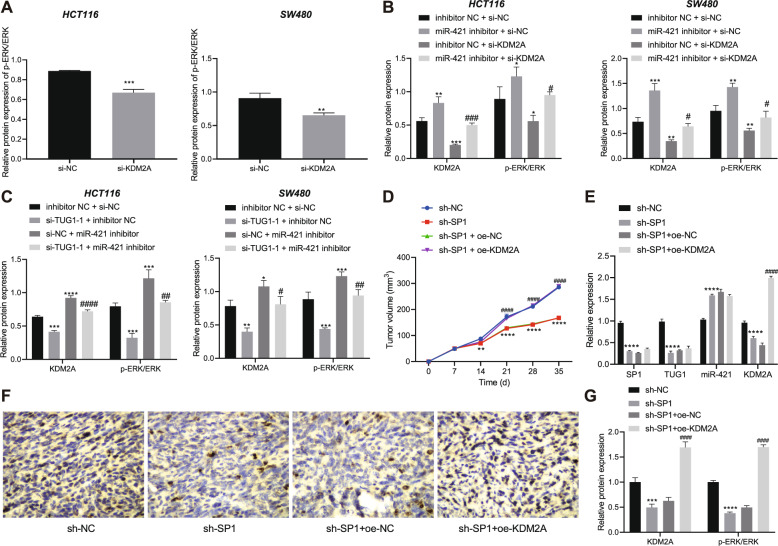
SP1 facilitates the tumorigenesis of CRC cells in vivo by regulating theTUG1/miR-421/KDM2A/ERK axis. **A** Western blot assay for p-ERK and ERK protein levels in HCT116 and SW480 cells transfected with si-KDM2A. **B** Western blot assay for KDM2A, p-ERK and ERK protein levels in HCT116 and SW480 cells transfected with miR-421 inhibitor, si-KDM2A or both. **C** Western blot assay for KDM2A, p-ERK, and ERK protein levels in HCT116 and SW480 cells transfected with si-TUG1-1, miR-421 inhibitor, or both. **D** Tumor volume in nude mice treated with sh-SP1 or combined with oe-KDM2A at different time points. **E** RT-qPCR determination of SP1, TUG1, miR-421, and KDM2A expression in the tumor tissues of mice treated with sh-SP1 or combined with oe-KDM2A. **F** Immunohistochemical staining of KDM2A protein in tumor tissues of mice treated with sh-SP1 or combined with oe-KDM2A. **G** Western blot assay of KDM2A, p-ERK, and ERK expression in tumor tissues of mice treated with sh-SP1 or combined with oe-KDM2A. ^*^p < 0.05, ^**^p < 0.01, ^***^p < 0.001, and ^****^p < 0.0001, compared with HCT116 and SW480 cells transfected with si-NC or inhibitor NC + si-NC, or mice treated with sh-NC. ^#^*p* < 0.05, ^##^*p* < 0.01, ^###^*p* < 0.001, and ^####^*p* < 0.0001, compared with HCT116 and SW480 cells transfected with miR-421 inhibitor + si-NC or si-TUG1-1 + inhibitor NC, or mice treated with sh-SP1 + oe-NC. The measurement data are expressed as mean ± standard deviation. Comparison between two groups was conducted by unpaired *t*-test. Multi-group comparison at different time points was performed using two-way ANOVA, followed by Bonferroni post hoc test, *n* = 12.

We further investigated the role of miR-421-mediated KDM2A inhibition in regard to TUG1-induced activation of ERK pathway in CRC. First, we knocked down KDM2A in HCT116 and SW480 cells. Western blot assay results revealed that the ratio of p-ERK/ERK was decreased in HCT116 and SW480 cells with KDM2A knockdown (Fig. 6A). Next, HCT116 and SW480 cells were transfected with miR-421 inhibitor, si-KDM2A or both. The results of western blot assay suggested that miR-421 inhibition led to an increase in the ratio of p-ERK/ERK and conversely, si-KDM2A or miR-421 inhibitor + si-KDM2A reduced the ratio of p-ERK/ERK (Fig. 6B and Supplementary Fig. 7A).

For further investigation, HCT116 and SW480 cells were transfected with si-TUG1-1, miR-421 inhibitor or both. Western blot assay results demonstrated that TUG1 silencing could reduce the protein levels of KDM2A and the ratio of p-ERK/ERK, while miR-421 inhibitor or si-TUG1-1 + miR-421 inhibitor resulted in elevation in the protein levels of KDM2A and the ratio of p-ERK/ERK (Fig. 6C and Supplementary Fig. 7B). The above results indicated that TUG1 up-regulated the expression of KDM2A by inhibiting miR-421 and thus activated the ERK pathway.

Lastly, we performed xenograft tumor in nude mice to evaluate the effect of SP1 on the tumorigenesis of CRC cells in vivo through regulation of the TUG1/miR-421/KDM2A/ERK pathway (Supplementary Fig. 8). SP1 silencing suppressed the tumor growth, which was reversed by overexpression of KDM2A (Fig. 6D). What’s more, SP1 knockdown caused a reduction in the expression of SP1, TUG1, and KDM2A along with the ratio of p-ERK/ERK yet an increase in the expression of miR-421. In contrast, further overexpression of KDM2A increased the expression of KDM2A and the ratio of p-ERK/ERK (Fig. 6E–G). Overall, these results indicated that SP1 up-regulated TUG1, and consequently inhibited miR-421, increased KDM2A and activated the ERK pathway, thus promoting the tumorigenesis of CRC cells in vivo.

## Discussion

**Fig. 7:**
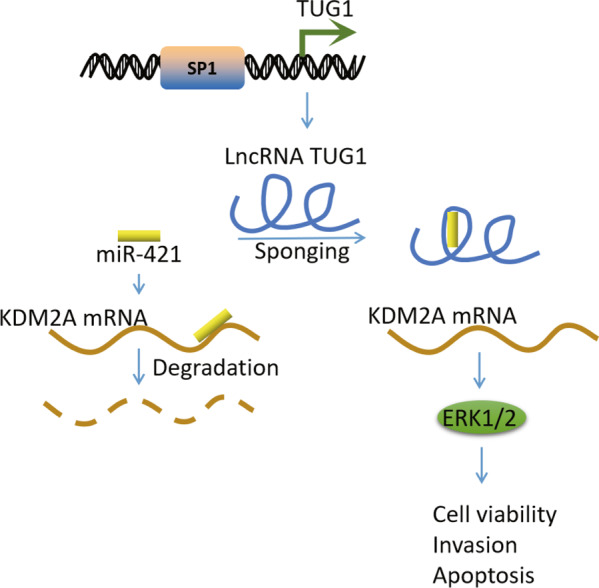
Schematic diagram of the SP1-mediated oncogenic mechanism in CRC progression. TUG1, positively regulated by SP1, could bind to miR-421 to up-regulate KDM2A, thereby activating the ERK pathway and consequently enhancing CRC cell viability and invasion while repressing cell apoptosis.

Based on the reported interaction between SP1 and TUG1 [13], the current study set out to explore this interaction in regard to CRC, and uncovered that TUG1, upon activation by transcription factor SP1, could bind to miR-421 to up-regulate KDM2A expression and activate the ERK pathway, consequently facilitating the progression of CRC (Fig. 7). The first major findings in our study were the up-regulated expression of TUG1 in CRC tissues and cells, whereas silencing of TUG1 led to suppression of CRC cell malignant potentials. Dysregulation of TUG1 has also been previously documented in other studies, and even associated with diverse physiological processes, including tumorigenesis [22, 23]. In addition, loss of TUG1 is known to markedly impede the migration ability of CRC cells [24]. Furthermore, a previous study has demonstrated the ability of TUG1 to mediate epithelial-mesenchymal transition (EMT) markers and potentiate the metastatic potential of CRC cells [25]. Additionally, TUG1 knockdown can reverse EMT to restrain the proliferative, migration, and invasive potentials of CRC cells, which is very much in line with our findings [26]. Meanwhile, TUG1 is regarded as a downstream molecular of TGF-β, with evidence even suggesting that TUG1 may function as a pro-metastatic lncRNA responsible for the TGF-β-induced metastasis in CRC [27]. In addition to the tumor-promoting function of TUG1, our findings further identified SP1, which was an up-regulated gene in CRC, to serve an upstream mediator of TUG1. Numerous studies have also documented this oncogenic property of SP1 in CRC, which underscores the critical relevance of our findings [28, 29]. Besides, the regulatory role of SP1 in another lncRNA has been discovered, whereby SP1 functions as a transcription factor to up-regulate ZFAS1 expression, thus expediting the progression of CRC [30].

Additionally, the current study further demonstrated the downstream modulatory axis miR-421/KDM2A to be linked with CRC progression. Notably, a previous study highlighted that TUG1 could bind to miR-26a, and further negatively regulated its expression to accelerate the progression of prostate cancer [31]. More importantly, TUG1 is known to augment the tumorigenesis in CRC by controlling an axis consisting of miR-26a-5p/MMP14/p38 MAPK/Hsp27, which is involved in the oncogenic process [32]. Similarly, TUG1 has been reported to function as a ceRNA of miR-197-3p, wherein knockdown of TUG1 could retard disease progression and enhance the sensitivity of CRC cells to 5-fluorouracil, reiterating the significance of TUG1 silencing in CRC [33]. TUG1 might also act as a ceRNA of miR-186 and impair miR-186-mediated inhibition of CPEB2 to strengthen methotrexate resistance in CRC [34]. Providing further insight, the study performed by Sun et al. [8] proposed the blockade of miR-600-caused down-regulation of KIAA1199 as the mechanism underlying the pro-metastatic effect of TUG1 on CRC cells. Meanwhile, a different pro-metastatic mechanism in CRC was also recently illustrated, such that TUG1 could bind to miR-153-1 and down-regulate KLF4, a transcription factor of miR-153-1, by recruiting EZH2 [35]. The aforementioned findings indicate the involvement of diverse molecular mechanisms in the different biological functions of TUG1. Expanding on our current information, our findings further displayed that TUG1 could bind to miR-421 to up-regulate the expression of KDM2A, a target gene of miR-421. Consequently, it would be plausible to suggest that miR-421 can down-regulate KDM2A, contributing to the promotion of CRC cellular apoptosis and repression of cell growth. Consistent with our findings, miR-421 was previously highlighted as a tumor-suppressive miRNA that restrains the malignant characteristics of CRC cells [15], while KDM2A is known to serve as a cyclin D1-associated mediator facilitating cell proliferation and colony formation in colorectal adenocarcinoma [17]. Collectively, our findings and existing data indicate that TUG1 can bind to miR-421 to up-regulate KDM2A, thus expediting CRC cell growth and invasion.

Furthermore, subsequent findings in our study revealed that TUG1 up-regulated KDM2A to induce the activation of the ERK pathway. In addition, the results of gain-of-function and rescue experimentation indicated that inhibition of the ERK pathway reversed the pro-invasive and anti-apoptosis action of TUG1. Inherently, KDM2A is known to function as an oncogene in lung cancer via activation of the ERK pathway [36]. On the other hand, knockdown of TUG1 was recently demonstrated to impede the proliferative property of CRC cells and their tumorigenicity in in vivo settings by reducing the activity of the Wnt/β-catenin pathway [10]. Meanwhile, published literature has further shown the involvement of the ERK pathway in the EGF-induced inhibition of E-cadherin that can stimulate CRC cell migration [37], which highlights the oncogenic role of ERK activation in CRC [38]. Consequently, it seems reasonable to conclude that TUG1 could activate the ERK pathway, thereby contributing to tumorigenesis.

Collectively, findings uncovered in the current study unravel a regulatory network implicated in the pathogenesis and progression of CRC, wherein SP1 up-regulates TUG1, which activates the ERK pathway via miR-421-induced down-regulation of KDM2A. In addition, silencing of TUG1 was demonstrated to potentially impede the growth of CRC cells while stimulating their apoptosis. Our findings therefore highlight that TUG1 may be a new therapeutic target for CRC.

## Supplementary information

Supplementary Materials

aj-checklist

## Data Availability

The datasets generated and/or analysed during the current study are available from the corresponding author on reasonable request.
