# Supplementary material for: SP1-mediated up-regulation of lncRNA TUG1 underlines an oncogenic property in colorectal cancer
Source: Cell Death Dis. 2022 May 4;13(5):433. doi: 10.1038/s41419-022-04805-w (PMC9068916; doi:10.1038/s41419-022-04805-w)
Supplement: Supplementary file 1 — Supplementary Materials [file 41419_2022_4805_MOESM1_ESM.pdf]

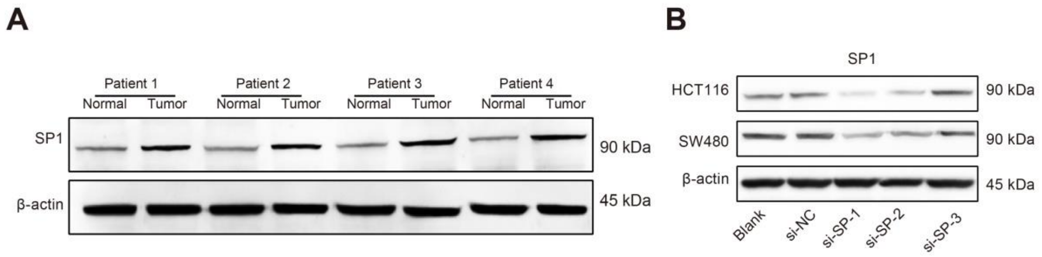

Supplementary Fig. 1 The representative Western blots of the panel C and F of Fig. 1.

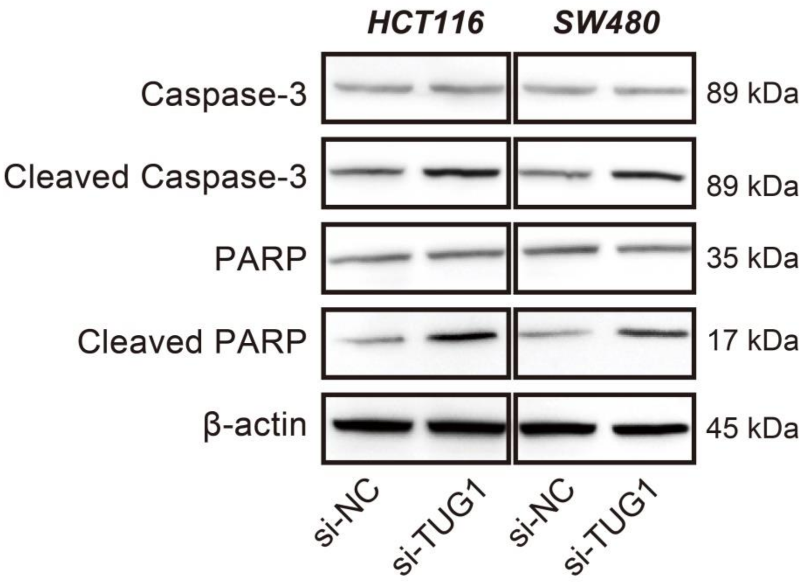

Supplementary Fig. 2 The representative Western blots of the panel E of Fig. 2.

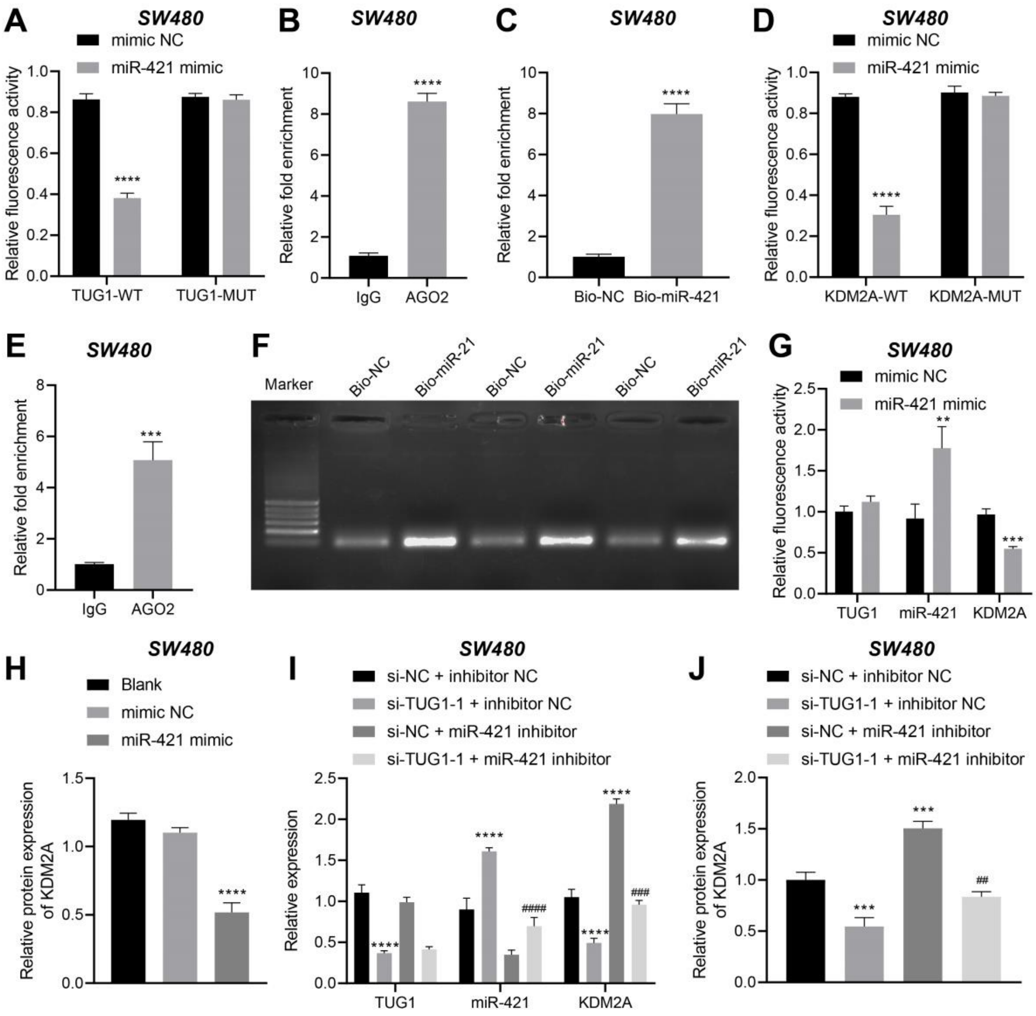

Supplementary Fig. 3 TUG1 positively regulates KDM2A by binding to miR-421 in SW480 cells. A: The binding of TUG1 to miR-421 in SW480 cells verified by dual-luciferase reporter assay. B: The binding of TUG1 to miR-421 in SW480 cells tested by RIP assay in combination with qPCR. C: The binding of TUG1 to miR-421 in SW480 cells tested using RNA pull-down. D: The binding between miR-421 and KDM2A in SW480 cells identified using dual-luciferase reporter assay. E: The binding of miR-421 to KDM2A in SW480 cells tested by RIP assay in combination with qPCR. F: The binding of miR-421 to KDM2A in SW480 cells tested using RNA pull-down. G: Determination of TUG1, miR-421 and KDM2A expression in SW480 cells transfected with miR-421 mimic by RT-qPCR. H: Measurement of KDM2A protein level in SW480 cells transfected with miR-421 mimic by Western blot assay. I: Determination of TUG1, miR-421 and KDM2A expression in SW480 cells transfected with si-TUG1-1 or combined with miR-421 inhibitor by RT-qPCR. J: Measurement of KDM2A protein level in SW480 cells transfected with si-TUG1-1 or combined with miR-421 inhibitor by Western blot assay. $^{**}p < 0.01$, $^{***}p < 0.001$ and $^{****}p < 0.0001$, compared with SW480 cells transfected with mimic NC or si-NC + inhibitor NC, or SW480 cells incubated with IgG or Bio-NC. $^{\#\#}p < 0.01$,

$^{\#\#\#}p < 0.001$, and $^{\#\#\#\#}p < 0.0001$, compared with SW480 cells transfected with si-TUG1-1 + inhibitor NC. The measurement data are expressed as mean ± standard deviation. The experiments were repeated three times independently. Comparison between two groups was conducted by unpaired *t*-test. Multi-group comparison was conducted by one-way ANOVA with Tukey's *post hoc* test.

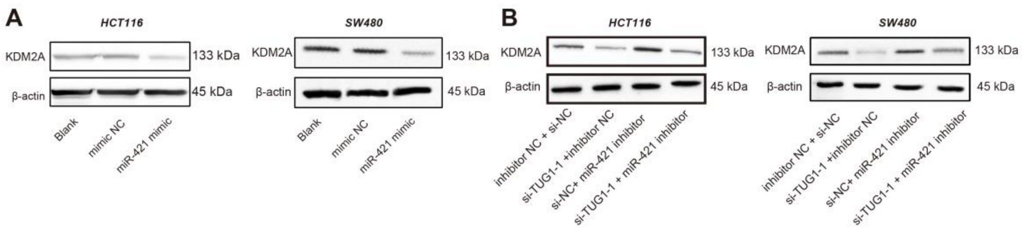

Supplementary Fig. 4 The representative Western blots of the panel K and M of Fig. 3, as well as panel H and J of Supplementary Fig. 3.

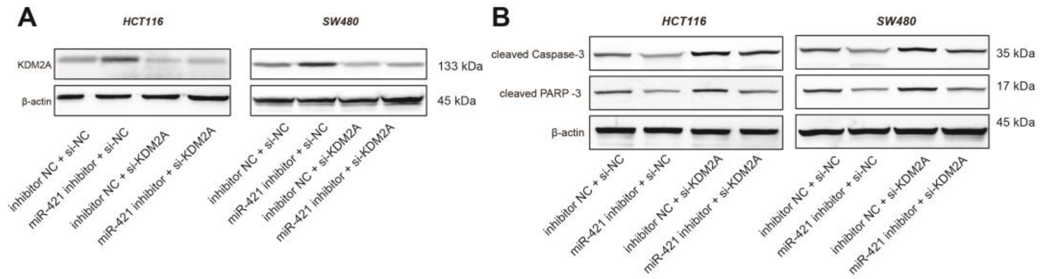

Supplementary Fig. 5 The representative Western blots of the panel B and F of Fig. 4.

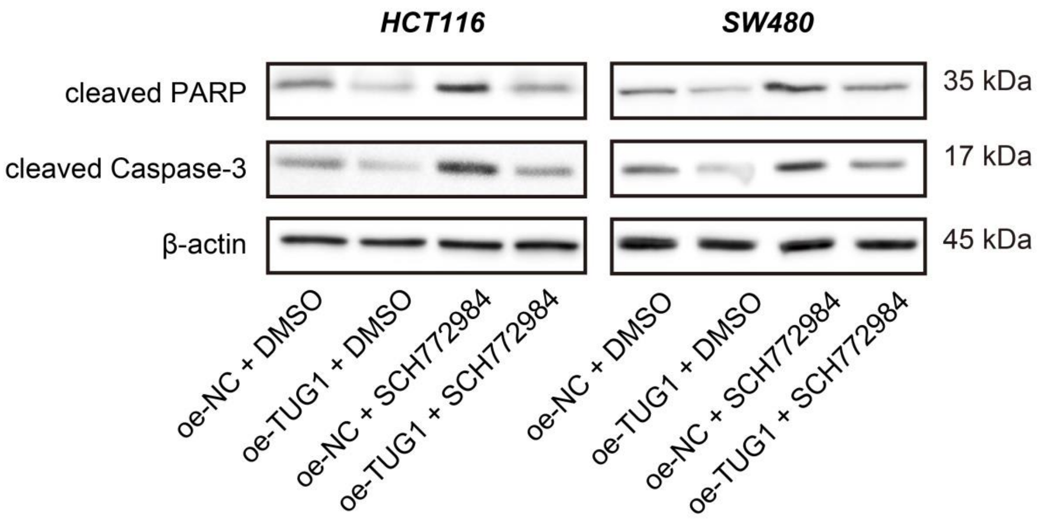

Supplementary Fig. 6 The representative Western blots of the panel E of Fig. 5.

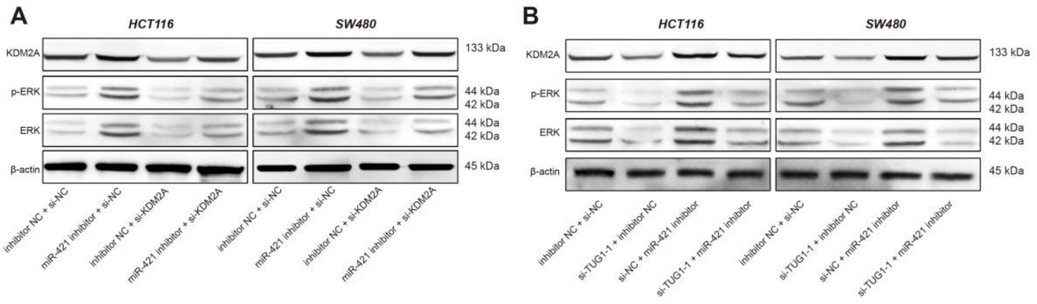

Supplementary Fig. 7 The representative Western blots of the panel B and C of Fig. 6.

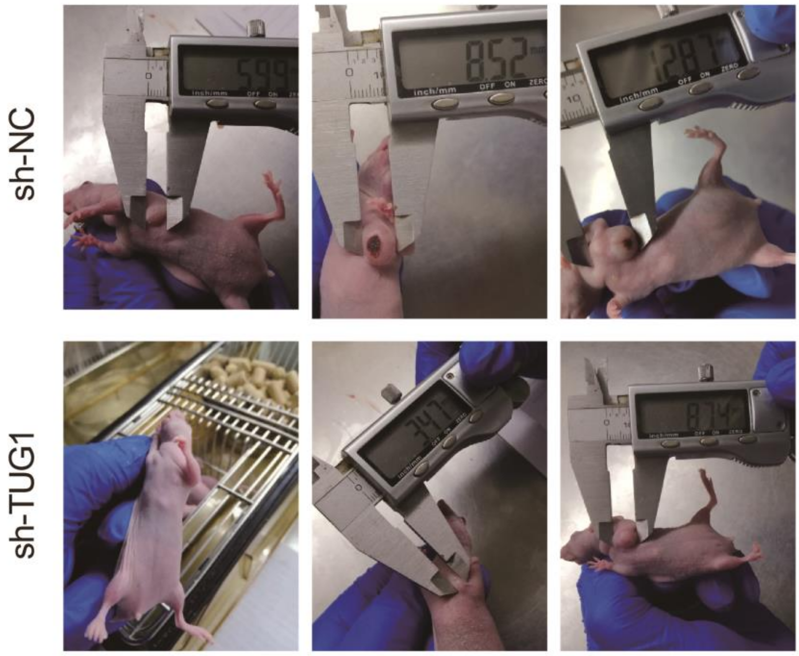

Supplementary Fig. 8 Measurement of long and short diameter of nude mice bearing tumors.

Supplementary Table 1 Sequences of siRNAs

siRNA	Forward (5'→3')	Reverse (5'→3')
si-SP1-1	CAGAUACCAGACCUCUUCU	AGAAGAGGUCUGGUAUC UG
si-SP1-2	CCAACAGAUUAUCACAAAU	UUGUUUGUCUGAUCGGCUCTT
si-SP1-3	UGAUCUGCCUCUCAACUGCCC	UACCGGGAAACUGGAGCACU
si-TUG1-1	GGCUGAUGCAUAUGGUACATT	UGUACCAUAUGCAUCAGCCTT
si-TUG1-2	CCCUCCAUGAAUACCUGAATT	UUCAGGUAUUCAUGGAGGGTT
si-KDM2A	GAACCCGAAGAAGAAAGGAUUCGUU	AACGAAUCCUUUCUUCUUCGGGUUC

Supplementary Table 2 Sequences of RT-qPCR

Genes	Forward (5’→3’)	Reverse (5’→3’)
SP1	CCATACCCCTTAACCCCG	GAATTTTCACTAATGTTTCCCACC
TUG1	TAGCAGTTCCCCAATCCTTG	CACAAATTCCCATCAT TCCC
KDM2A	TCCCCACACACATTTTGACATC	GGGGTGGCTTGAGAGATCCT
miR-421	CTCACTCACATCAACAGACATTAATT	TATGGTTGTTCTGCTCTCTGTGTC
U6	GGAACGCTTCACGAATTTG	ATTGGAACGATACAGAGAAGATT
GAPDH	GGACCTGACCTGCCGTCTAG	GTAGCCCAGGATGCCCTTGA

Supplementary Table 3 Correlation of TUG1 expression with the clinicopathological characteristics of colorectal cancer patients

Variables	Cases	TUG1 expression		*p* value
		Low (n = 10)	High (n = 10)	
Age (years)				0.178
≥ 60	11	7	4	
< 60	9	3	6	
Gender				0.658
Female	9	5	4	
Male	11	5	6	
Tumor size				0.006
≥ 5 cm	8	1	7	
< 5 cm	12	9	3	
TNM stage				0.028
I	7	6	1	
II	6	3	3	
III	7	1	6	
LNM				0.019
Yes	7	1	6	
No	13	9	4	

Note: LncRNA, long non-coding RNA; TUG1, Taurine up-regulated gene 1; TNM, tumor node metastasis; LNM, lymph node metastasis.
